# Supplementary material for: Glomerular Filtration Rate in Former Extreme Low Birth Weight Infants over the Full Pediatric Age Range: A Pooled Analysis
Source: Int J Environ Res Public Health. 2020 Mar 24;17(6):2144. doi: 10.3390/ijerph17062144 (PMC7142917; doi:10.3390/ijerph17062144)
Supplement: Supplementary file 1 [file ijerph-17-02144-s001.pdf]

Table S1. Overview of the formulae applied to calculated the estimated or inulin derived glomerular filtration rate (eGFR) in the different studies.

Study	**Formulae as applied to calculate eGFR in the different studies**
Rodríguez-Soriano 2005 [12]	$0.45 \times \frac{heigt\ [cm]}{plasma\ creatinine\ [mg/dl]}$
Kleizer-Veen 2007 [13]	$U\ (mg/dL) \times \frac{V\ (mL)}{P\ (mg/dL)} \times duration\ of\ clearance\ period\ [min]$
Starzec 2016 [14]	$-4.32 + \frac{80.35}{Cystatin\ C}$
Yamamura-Miyazaki 2015 [15]	$104.1 \times \frac{1}{serum\ Cys\ C\ [mg/L]} - 7.80$<br><br>$110.2 \times \frac{reference\ sCr}{patient'sCr} + 2.93$
Raaijmakers 2017 [16]	$130 \times Cystatin\ C^{1.069} \times age^{0.117} - 7$<br><br>$0.413 \times \frac{height\ [cm]}{serum\ creatinine\ [mg/dl]}$
Vollsaeter 2018 [17]	$0.413 \times \frac{height\ [cm]}{serum\ creatinine\ [mg/dl]}$<br><br>$\frac{507.76 \times e^{0.003 \times height}}{Cystatin\ C^{0.635} \times Creatinine^{0.547}}$<br><br>$0.68 \times \frac{height\ [cm]}{serum\ Creatinine\ \left[\frac{mg}{dl}\right]} - 0.0008 \times \left(\frac{height\ [cm]}{serum\ Creatinine\ \left[\frac{mg}{dl}\right]}\right)^2 +$<br>$0.48 \times age\ [years] - (21.53\ in\ males\ or\ 25.68\ in\ females)$
Bachetta 2009 [19]	$U\ (mg/dL) \times \frac{V\ (mL)}{P\ (mg/dL)} \times duration\ of\ clearance\ period\ [min]$
Zaffanello 2010 [20]	$0.413 \times \frac{height\ [cm]}{serum\ creatinine\ [mg/dl]}$<br>$39.1 \left(\frac{height\ (m)}{SCr\ (mg/dl)}\right)^{0.516} \times \left(\frac{1.8}{Cystatin\ C\ (mg/L)}\right)^{0.294}$<br>$\times \left(\frac{30}{BUN\ (mg/dl)}\right)^{0.169} (1.099)^{male} \times \left(\frac{height\ (m)}{1.4}\right)^{0.188}$
Matsumura 2019 [21]	$110.2 \times \frac{reference\ sCr}{patient'sCr} + 2.93$
